# MoO_3_ Nanobelts Synthesized from Recycled Industrial Powder and Applied as Electrodes for Energy Storage Applications

**DOI:** 10.3390/nano15171380

**Published:** 2025-09-08

**Authors:** Angelo Di Mauro, Federico Ursino, Giacometta Mineo, Antonio Terrasi, Salvo Mirabella

**Affiliations:** 1 Dipartimento di Fisica e Astronomia “Ettore Majorana”, Università degli Studi di Catania, Via S. Sofia 64, 95123 Catania, Italy; angelo.dimauro@dfa.unict.it (A.D.M.); federico.ursino@dfa.unict.it (F.U.); giacometta.mineo@dfa.unict.it (G.M.); antonio.terrasi@dfa.unict.it (A.T.); 2 CNR-IMM, Università di Catania, Via S. Sofia 64, 95123 Catania, Italy

**Keywords:** α-MoO_3_ nanobelts, recycled industrial waste, hydrothermal synthesis, energy storage, supercapacitors, electrochemistry, specific capacitance, alkaline electrolytes

## Abstract

The sustainable development of our society faces significant challenges, including the need for environmentally friendly energy storage devices. Our work is concerned with the conversion of Mo-based recycled industrial waste into active nanocatalysts for energy storage applications. To reach this goal, we employed hydrothermal synthesis, a low-cost and temperature-scalable method. The proposed synthesis produces MoO_3_ nanobelts (50–200 nm in width and 2–5 µm in length) with a high yield, about 74%. The synthesized nanostructures were characterized in 1 M KOH and 1 M NH_4_OH, as alkaline environments are a promising choice for the development of eco-friendly devices. To investigate the material’s behaviour cyclic voltammetry (CV), galvanostatic charge–discharge (GCD), and electrochemical impedance spectroscopy (EIS) measurements were carried out. From CV curves, it was possible to evaluate the specific capacitance values of 290 and 100 Fg^−1^ at 5 mVs^−1^ in 1 M KOH and 1 M NH_4_OH, respectively. Also, GCD was employed to evaluate the specific capacitance of the material, resulting in 75 and 60 Fg^−1^ in 1 M KOH and 1 M NH_4_OH, respectively. CV and GCD analyses revealed that MoO_3_ nanobelts act as two different types of energy storage devices: supercapacitors and pseudocapacitors. Additionally, EIS allowed us to distinguish between the resistive and capacitive behaviour contributions depending on the electrolyte. Furthermore, it provided a comprehensive electrochemical characterization in different alkaline electrolytes, with the intention of conjugating waste management and sustainable energy storage device production.

## 1. Introduction

The development of our society also involves the research of sustainable energy storage solutions with superior performance; for instance, new energy technologies are required for the automotive field, mobile electronics, and many other applications [1,2].In addition, the high environmental impact of classic Li-ion batteries is one of the reasons that justify the study of new paths for energy storage [3,4].

New electrochemical energy storage (EES) devices with a low environmental impact have been studied in the last few decades, and these devices can be divided into different types with respect to their performances and active storage mechanisms [5]. Comprehensive knowledge of the relationship between materials and electrolytes in EES devices is a crucial point in this field [6]; hence, superior comprehension about storage mechanisms can open new routes to improve electrochemical device performance [7].

Many materials are eligible for energy storage applications, but among them, transition metal oxides (TMOs) are promising materials; indeed, their stability and the presence of many oxidation states are favourable for these applications [8]. Their characteristics also allow for their employment in cutting-edge applications like flexible supercapacitors, as studied by Delbari et al. [9]. Today, not all TMOs are favourable for use in these applications; indeed, for each material, costs and the social impact of their production are considered; hence, the European commission has published a list of critical raw materials (CRMs) [10]. Among transition metals, there is molybdenum (Mo), which is a favourable material because it is not a CRM and due to its interesting performance in energy storage applications [11,12].

The shift to a circular economy has been much discussed in the last few years [13], and for this reason, many researchers are studying solutions to recover materials from waste, as demonstrated by Kim et al. in their work in which they directed their attention toward recovering materials in hydrometallurgical processes [14]; or in the work of Aloraini and Saees, which focused on recycling Pb from exhausted lead-acid batteries to produce polymeric-based materials [15]. Recycled material can be the starting point for the development of new technologies, as was carried out in our previous work. This consideration of waste as a resource is labelled “urban mining”, and it is a good practice that well-accepted by many scientists today, especially regarding e-waste materials [16]. In this context, the possibility to develop materials that can easily be recycled is also important, as shown by Lin et al. [17].

A key role in this scenario is taken by nanostructures; their surface–volume ratio is favourable to deliver high performance and a low employment of material with respect to applications with bulk materials [18,19]. Thanks to their unique shape and size, different effects can be seen, like quantum confinement effects and highly altered surface energy, modifying the bond structures close to the material’s surface [20,21]. To evaluate the performance of MoO_3_, the possibility of nanostructuring it to reveal the optimal ratio between structure and performance is of particular importance. A huge number of structures with controllable dimensions or morphologies can be obtained, starting from the zero-dimensional quantum dots (QDs) prepared by Lu et al., using intercalation and thermal exfoliation techniques [22], one-dimensional nanowires [23] or nanobelts [24,25,26], such as those synthesized by Jiang et al., starting from sodium molybdate (Na_2_MoO_4_) as a molybdenum source and NaCl as a capping agent, which show promising supercapacitive performance [27], two-dimensional nanoflowers, which are used for sensing thanks to their large active surface [28], and three-dimensional nanospheres that are also used in the field of catalysts [29].

Thanks to their promising characteristics and unique structure, MoO_3_ nanobelts are used as active materials in supercapacitors, especially in alkaline electrolytes such as KOH. In fact, many research groups, such as K. Gosh et al., have demonstrated a specific capacitance of 136 Fg^−1^ in a 6 M KOH electrolyte in a voltage window ranging from 0 to 1 V [30]. MoO_3_ nanobelts were synthesized and characterized by X. Zhang et al. in a three-electrode electrochemical cell using a standard calomel electrode as a reference, a platinum wire as a counter, nickel foam as a substrate for the MoO_3_ deposited active material, and 3 M KOH as the electrolyte. They obtained a specific capacitance of 206 Fg^−1^ at 1 mV s^−1^, with a potential range of 0 to 0.65 V [31].

Therefore, the aim of this work is to synthesize MoO_3_, which is a TMO, with a nanobelt morphology to study the base processes in energy storage. For this reason, at this starting point, no carbon black or other conductive additives that would significantly improve the performance of the electrode material were added.

The starting materials consisted of recycled industrial waste, and the final application is in the energy storage field; for this reason, we studied the behaviour of the material in different electrolytes. The material was widely characterized using different techniques, such as scanning electron microscopy, X-ray diffraction, Raman spectroscopy, and UV–vis spectrophotometry. Indeed, our goal is to open the route for the use of recycled material as a new resource for the development of new devices. Also, a deeper understanding of the energy mechanisms that govern the material was obtained using different electrochemical analysis techniques, such as cyclic voltammetry, galvanostatic charge–discharge, and electrochemical impedance spectroscopy, to evaluate the behaviour of the material. The results are also followed by the synthesis method; it is a novel synthesis method, which uses Mo-based recycled powder from industrial waste as a starting material for the process. This work can open new paths that conjugate a deeper knowledge of energy mechanisms and valorization of industrial waste.

## 2. Materials and Methods

### 2.1. Synthesis of MoO_3_-Nanobelts

**Figure 1:**
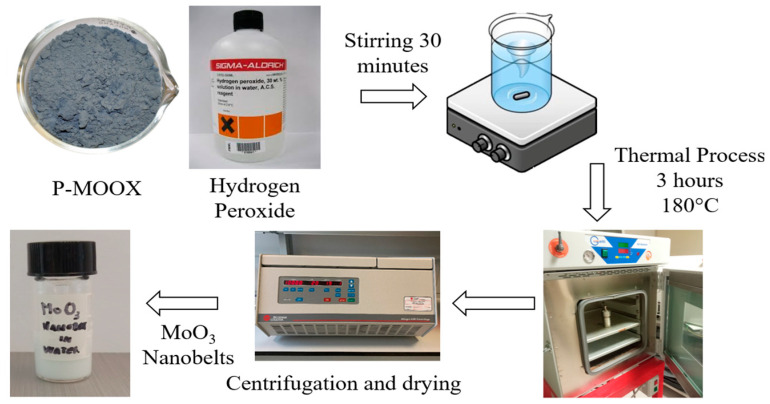
The synthesis process of MoO_3_ nanobelts started with mixing P-MOOX with hydrogen peroxide, where an exothermic reaction occurs. Then, the resulting solution was magnetically stirred for 30 min and transferred to a Teflon-lined container, which was placed in an oven for 3 h at 180 °C. After the hydrothermal treatment, the solution was centrifugated and dried, and the resulting powder containing ${MoO}_{3}$ nanobelts was dispersed in water.

The process that led to the synthesis of α-MoO_3_ nanobelts began with molybdenum-based recycled powder, obtained from Spirit S.R.L. (Chiampo, Vicenza, Italy), recovered from industrial waste. This route was specifically adopted for the case of our initial recycled starting powder. The company has developed a recovery process for this waste, which begins with an inertization to eliminate toxic gases, followed by a heat treatment at 700 °C for 3 h. At the end of this thermal process, the product is shredded to obtain powders, commercially known as recycled P-MOOX. A two-steps synthesis method, as shown in Figure 1, was used to produce the nanobelts. The first step was the exothermic reaction between 0.5 g of recycled P-MOOX and 5 mL of hydrogen peroxide (H_2_O_2_, %30 wt, Sigma Aldrich, Saint Louis, Missouri, USA), which led to the formation of a peroxo-molybdate solution, which is an unstable acidic solution. In addition, a further 4 mL of H_2_O_2_ was added, and the produced peroxo-molybdic acid solution was magnetically stirred for 30 min, and then the pH of the solution was measured; the recorded value was 1.26.

The second step concerned hydrothermal treatment. The final peroxo-molybdic acid solution was poured into a Teflon-lined container, and a volume of 25 mL was obtained by adding deionized water.

The Teflon-lined container was placed in an autoclave and then placed in a muffle for 3 h at 180 °C, and it was subsequently cooled at room temperature for 18 h. After the thermal treatment, the solution was centrifuged at 6000 rpm for 30 min, then washed with ethanol and deionized water, and finally dried on a hot plate heated to 75 °C for 45 min. The dried MoO_3_ nanobelt powder produced in this way was labelled as MoO_3_ NBs. Starting from 0.5 g of recycled P-MOOX, we obtained about 0.37 g of MoO_3_ NBs, giving a yield of about 74%.

### 2.2. Electrode Preparation

**Figure 2:**
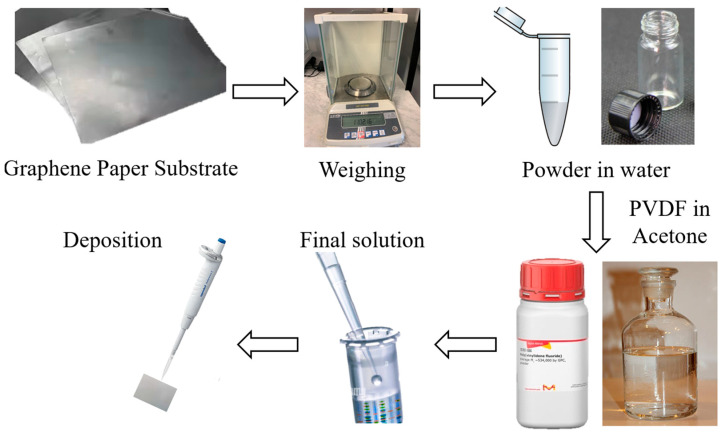
The preparation of the electrode started by taking graphene paper as a substrate, with an area of 1.5 cm^2^, and the bare substrate was weighed. Then, a mixture of MoO_3_ nanobelts and PVDF in acetone was deposited onto the substrate. Once dried, the electrode was weighed again, and the difference between the two recorded masses was assigned to the presence of the nanostructures.

The MoO_3_-based electrode was prepared starting from a graphene paper substrate (GP, Sigma Aldrich, 1 × 1.5 cm^2^, 240 μm thick), in which the solution containing the MoO_3_ nanobelt powder was deposited by drop-casting (approximately 40 μL of solution, which was left to dry completely for a day). The solution consisted of nanobelt powder dispersed in deionized water, with 1 mg of the first in 1 mL of the second, with the addition of 50 μL of polyvinylidene difluoride (PVDF, purchased by Sigma Aldrich) in acetone (concentration 11 gL^−1^) as a binder, which conferred good adhesion of the material to the substrate, preventing the active material detaching from the electrode once immersed in solution. Furthermore, the presence of the binder does not affect the performance of nanobelts, as shown in the literature [32]. The obtained electrode was weighed using a microbalance (Mettler Toledo MX5, Columbus, Ohio, USA) with a sensitivity of 0.01 mg, and the final mass of the electrode was estimated by evaluating the difference between the weight of the obtained electrode and of the bare GP substrate. All the steps are reported in Figure 2. The exposed area and the mass deposited were in a ratio of about 1 mg cm^−2^; in particular, the mass deposited on the electrode was about 0.2 mg and covered an area of about 0.2 cm^2^. In conclusion, in making this electrode, only our synthesized material and PVDF were used, and no carbon black was added.

### 2.3. Characterization Techniques

To investigate the morphology of the nanobelts, a Gemini field emission SEM Carl Zeiss SUPRATM 25 scanning electron microscope (SEM) (FEG-SEM, Carl Zeiss Microscopy GmbH, Jena, Germany) was used. The microscope was set to the immersion lens (in-Lens) mode. To define the crystal structure, X-ray diffraction (XRD) analysis was performed, and spectra were acquired by using a Bruker-AXSD5005θ-θ diffractometer (Billerica, Massachusetts, USA), with the use of Cu Kα radiation operating at 40 kV and at 30 mA. The XRD pattern was used to evaluate the d-spacing, which was calculated using Bragg’s law [33]:

(1) $$n\;\lambda =2\;d\;sin\theta \;=>\;d=\;\frac{n\lambda }{2sen\theta }$$

where λ = 1.5406 Å, θ is the peak position, and n is the diffraction order.

A PerkinElmer UV/Vis/NIR Lambda 1050+ spectrometer (Waltham, Massachusetts, USA) was used between 200 and 800 nm in 1 nm steps to acquire the transmittance spectrum of the sample. These data were employed to evaluate the optical bandgap using the interband absorption theory, according to the following equation [34,35]:

(2) $${\left(\;\alpha \;t\;h\;\nu \;\right)}^{n}=h\;\nu -\;E_{g}$$

where the absorption coefficient is α, h is Planck’s constant, t is the thickness of the film, ν is the light frequency, E_g_ is the optical bandgap energy, and the exponent n is a number which distinguishes the transition process, which can be equal to 2 or ½, respectively, for direct and indirect bandgaps. The extrapolated intercept of the linear region of the spectrum with the x-axis gives us an estimation of the optical bandgap

Information about the chemical structure was obtained by Raman analysis, performed using a Horiba scientific instruments model 1024X256-OE and with a THORLABS HNL225R laser with a 633 nm wavelength.

Energy storage performance was evaluated through electrochemical measurements by using a potentiostat (VersaSTAT 4 Princeton Applied Research, Oak Ridge, Tennessee, USA) with a three-electrode cell configuration with Ag/AgCl saturated in 3.5 M KCl as the reference electrode, the nanobelt electrode as the working electrode, and a platinum wire as the counter electrode. Cyclic voltammetry (CV) at different scan rates and galvanostatic charge–discharge (GCD) at different current densities were acquired in two different basic electrolytes, 1 M KOH and 1 M NH_4_OH, to evaluate the electrochemical performance of the MoO_3_ NBs from −0.6 to 0.1 V vs. the Ag/AgCl electrode. From the CV curves, the specific capacitance $C_{s}$ at each scan rate was evaluated using the following equation [36,37]:

(3) $$C_{s}=\frac{\int IdV}{m\nu ∆V}$$

where I is the current, m is the deposited mass, ν is the scan rate, and ∆V is the potential range. Dunn’s method was employed on the CV curves, thanks to it a surface-capacitive estimation, which was performed using the following formula [38]:

(4) $$i\cdot \;\nu^{-\frac{1}{2}}=k_{1}\nu^{-\frac{1}{2}}+k_{2}$$

where i is the current, ν is the scan rate, and $k_{1}$ and $k_{2}$ are parameters which refer to the surface and diffusion capacitive processes, respectively. The GCD analyses were performed in the same CV potential range with different current densities of 0.7, 1, 3, and 5 A g^−1^, and the discharge curves were studied to extract a more accurate estimate of the specific capacitance values whilst varying the current density, according to the following equation [39]:

(5) $$C_{s}=\frac{2I}{m{∆V}^{2}}\int_{V_{i}}^{V_{f}}V(t)\;dt$$

where $C_{s}$ is the specific capacitance, I is the current, m is the mass of the electrode, $t_{d}$ is the discharge time, ∆V is the range potential, and $\int_{V_{i}}^{V_{f}}V(t)\;dt$ is the area under the discharge curve of the GCD, which takes into account the possible non-linearity of the curve [38].

Electrochemical impendence spectroscopy (EIS) measurements were carried out in a potentiostatic mode through an AC voltage of 10 mV at an amplitude and frequency ranging from 100,000 to 0.1 Hz and at a potential 0 V vs. open circuit for both the electrolytes.

## 3. Results and Discussions

### 3.1. Morphological Characterization

**Figure 3:**
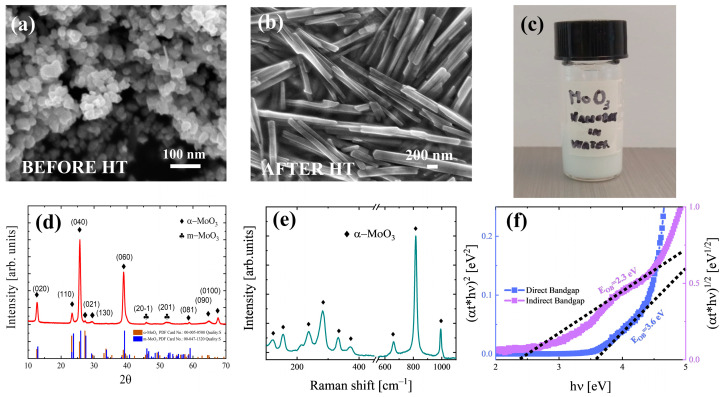
SEM images of (**a**) Mo-based starting powder and (**b**) MoO_3_ nanobelts at high magnification; (**c**) images of the MoO_3_ nanostructures dispersed in a water solution; (**d**) the red curve reports the XRD pattern of the resulting material, while blue and brown bars refer to the PDF cards of orthorhombic and monoclinic MoO_3_; (**e**) Raman spectrum of MoO_3_ nanobelts (light blue curve); (**f**) Tauc plots to evaluate direct (blue curve) and indirect (purple curve) bandgaps.

Figure 3a and Figure S1 show the Mo-based starting powder at high magnification and low magnification, respectively. The starting powder is characterized by a dominance of spheres with an average diameter of 5 µm. After the two-step synthesis process, the morphology drastically changes, as shown in Figure 3b, where nanostructures with smooth facets enclosed on the surfaces can be seen. In addition, the material forms agglomerated bundles. In situ measurements were carried out using the microscope software to determine the mean length and width of the nanobelts, showing an average length of 2 μm and width of 150–230 nm in the as-synthetized case, as reported in Figure S2; hence, the material can be described as having a nanobelt-like morphology. As these results show, it is observed that during the MoO_3_ nanobelt synthesis, different exothermic reactions occurred that led to the formation of several peroxo-molybdate species. These species are unstable, so they evolved into molybdic acid and then into the MoO_3_ nanobelts [15]. The reason of the formation of the nanobelts is also due to the acidic pH value that was reached during the synthesis, as reported in the literature, confirming the role of H^+^ ions in forming orthorhombic MoO_3_ nanobelts from the conversion of peroxo-molybdic species [40,41]. The nanostructures were stored in deionized water to prepare the sample for further analysis (see Figure 3c).

Figure S3 shows the XRD pattern of the initial P-MOOX powders. The peak positions are labelled, showing the presence of MoO_2_ and metallic molybdenum, as was also confirmed in previous work [41]. In Figure 3d, the XRD pattern of the MoO_3_ nanobelt sample is reported, and different peaks are present. These peaks were labelled assuming an orthorhombic α-MoO_3_ and a monoclinic β-MoO_3_ crystal structure. The X-ray diffraction data are reported in Table S1 compared with The International Center for Diffraction Data (ICDD) papers No. 00-047-1320 (monoclinic) and No. 00-005-0508 (orthorhombic). The presence of peaks of other phases or impurities were not detected by XRD.

As can be seen from Table S1, the MoO_3_ sample shows defined crystal structures, even if two different phases may be present: orthorhombic MoO_3_ (12.76°, 23.33°, 25.70°, 27.34°, 38.98°, 45.74°, 45.90°, 52.04°, 52,37°, 58,81°, 61.62°, 64.93°, and 69.47 2θ peaks, from ICDD Card No.: 00-005-0508) and monoclinic MoO_3_ (45.90° and 52.37° 2θ peaks from ICDD Card No. 00-047-1320). The [0k0] growth direction shows the most intense peaks, and this is the preferential growth direction of the nanobelts. The reason for this result is a consequence of surface energy modification along the other directions [42,43] due to the high concentration of H^+^ ions in the solution, as also confirmed by the low pH value. The presence of the H^+^ ions helps to saturate the dangling bonds along the other [h00] and [00l] directions. As shown by the data, the diffraction peaks along [020], [040], [060], and [0 10 0] have a stronger intensity compared to the standard ICDD data, indicating the orientation of nanostructures along that direction, thus resulting in anisotropic growth [44]. The d-spacing values for the dominance peaks were evaluated using Equation (1) (see Table S2), and an average value of about 3.5 Å was obtained.

Figure 3e shows the Raman spectra of the MoO_3_ sample. Around 600–1000 and 200–400 cm^−1^, the vibration modes in α-MoO_3_ appear. These correspond to the stretching, deformation, and lattice modes. In particular, the ones observed between 200 and 400 cm^−1^ correspond to bending modes of the orthorhombic crystal, while A_g_, B_1g_, B_2g_, and B_3g_ represent Raman-active modes. The peak at 991 cm^−1^ is due to the stretching mode of the terminal oxygen (Mo=O). The peak at 818 cm^−1^ is typical of the stretching mode of the doubly coordinated oxygen O (Mo=O). Finally, the peak at 663 cm^−1^ refers to the stretching mode of the triply coordinated oxygen (O-Mo-O) [45]. In conclusion, it is possible to say that the position of the peaks is in good agreement with those reported in the literature [44,46,47,48], while the sharpness of the peaks indicates an ordered structure [49], as also suggested by the XRD analysis.

The optical absorption coefficient of the sample was determined from the transmittance data (see Figure S4) using the Beer–Lambert law, and this was used to calculate direct and indirect bandgaps using Equation (2), as reported in Figure 3f, shown by the blue and purple curves, respectively. The resulting energy gaps are 3.6 eV for the direct allowed transitions and 2.3 eV for the indirect transitions. These values are close to those reported in the literature [44,46,50,51,52,53] for both cases in α-MoO_3_. It is possible that the presence, even in a small amount, of monoclinic β-MoO_3_, as observed in the XRD analyses, which has a bandgap of 4.15 eV [51], caused the slight variation in the expected value.

The executed analyses confirm the possibility of converting Mo-based recycled powder into α-MoO_3_ nanostructures. As suggested by the SEM images, our nanostructure has the typical shape and size of nanobelts, and XRD confirmed their preferential [0,k,0] growth direction and the main formation of the orthorhombic phase, which was also confirmed by the Raman spectrum, which was mainly characterized by the presence of peaks linked to the orthorhombic phase, and the values of the optical bandgap extracted by the UV–vis measurements are comparable with the orthorhombic phase

### 3.2. Electrochemical Characterization

**Figure 4:**
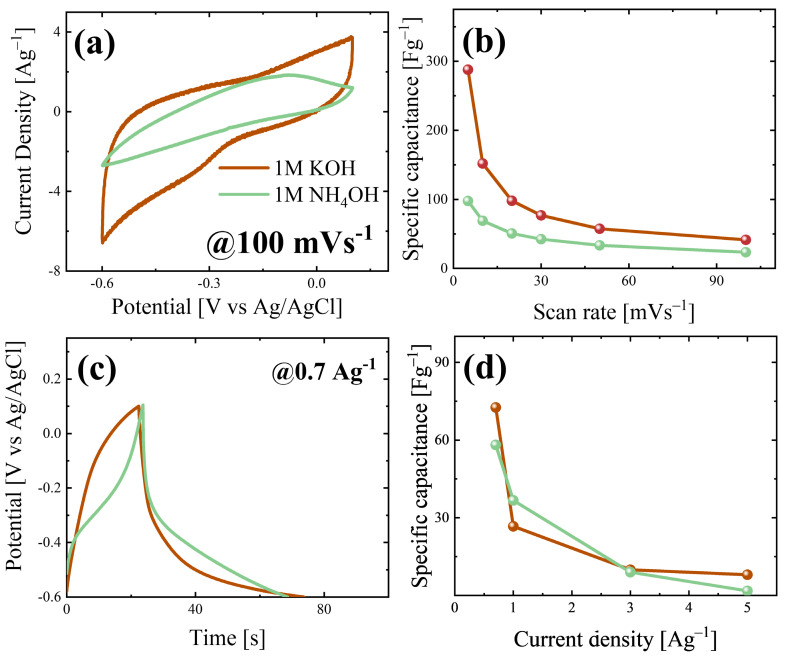
(**a**) CVs in 1M KOH (brown curve) and 1M NH_4_OH (green curve) at 100 mVs^−1^; (**b**) specific capacitance values extracted from CVs; (**c**) GCD curves of the sample in 1M KOH (brown curve) and 1M NH_4_OH (green curve) at 0.7 Ag^−1^; (**d**) Specific capacitance values calculated from GCD curves.

To evaluate the energy storage performance and mechanism of the MoO_3_ nanobelts, electrochemical analyses were performed in different electrolytes, i.e., 1 M KOH and 1 M NH_4_OH. Figure 4a reports the CV curves at 100 mVs^−1^ acquired using the same active material (MoO_3_ nanobelts) in 1 M KOH and 1 M NH_4_OH after several stability cycles (see Figure S5a,b), as shown by the brown and light green curves, respectively. In Figure S5c, the specific capacitance values with the increase in the cycle numbers are reported, showing good efficiency up to 30 cycles. The electrode tested in 1 M KOH gives the CV curve, whose shape is not linked to a specific behaviour; instead, the one obtained in 1 M NH_4_OH possesses a shape close to that of an ideal pseudocapacitor in these environments, with an oxidation peak around *−*0.2 V [4]. The comparison between the area under the CV curves suggests that the storage of K^+^ is favourable with respect to NH_4_^+^; indeed, the material shows a superior area for the CV in 1 M KOH compared to 1 M NH_4_OH. Furthermore, to confirm this experimental result, specific capacitance values were extracted from the CVs obtained at different scan rates and in both electrolytes (see Figure S6a,b) using Equation (3), and they are compared in Figure 4b. The MoO_3_ nanobelts show higher specific capacitance values in 1 M KOH, reaching about 290 Fg^−1^ at 5 mVs^−1^, while a specific capacitance of about 75 Fg^−1^ is obtained in 1 M NH_4_OH at the same scan rate. The expected superior performance in terms of the pseudocapacitive behaviour was not observed, and this was probably due to the corrosive effect of NH_4_OH on molybdenum-based oxides, which limits the electrochemical performance [54]. The electrochemical performance is not affected by the presence of m-MoO_3_, firstly due to the low amount present in the sample, and secondly because it has a structure that does not favour energy storage, while the orthorhombic one has a layered structure that favours energy storage processes [25]. At higher scan rates, the specific capacitance values decrease in both cases; this trend can be linked to not only the dominance of diffusive mechanisms but also to the presence of an electrical resistance for the sample, which limits the electrochemical performance. Dunn’s model was employed to estimate which type of mechanisms dominate when varying the electrolyte. Using Equation (4), an estimation for different scan rates was carried out for both 1 M KOH and 1 M NH_4_OH, as shown in Figure S7a and Figure S7b, respectively. The material in 1 M KOH showed a surface-capacitance contribution of about 35% at 5 mVs^−1^, and this was estimated at 49% for 1 M NH_4_OH. The same trend can be seen at higher scan rates, suggesting that in 1 M NH_4_OH, surface-capacitive mechanisms have superior weight with respect to 1 M KOH.

Figure 4c reports the galvanostatic charge–discharge (GCD) curves at 0.7 Ag^−1^ acquired for both the electrolytes, while Figure S8a,b report the curves at a higher current density. The sample tested in 1 M KOH shows a shape close to a quasi-triangular one, as seen for supercapacitors [55,56,57]. In 1 M NH_4_OH, there is a cusp-like shape, which is characteristic for pseudocapacitors; indeed, the change in the slope that occurred around *−*0.2 V is linked to oxidation reactions at the material’s surface; also, the shape of only the discharge curve suggests the presence of intercalation mechanisms with partial redox reactions [58]. The specific capacitance values were evaluated using Equation (5), and they are shown in Figure 4d, reporting values of 73 and 58 Fg^−1^ at 0.7 Ag^−1^, respectively, for 1 M KOH and 1 M NH_4_OH. The sample tested in 1 M NH_4_OH reports lower values at a high current density than the sample tested in the other electrolyte. This behaviour is usually linked to higher material resistance, which limits the storage mechanisms [59]. To validate this hypothesis, iR drop values were extracted for both electrolytes from the GCD curves (see Figure S8c), calculating the potential difference between the end and beginning of the charge and discharge curves, respectively. As expected, the iR drop increases with the current density, but the values obtained for the sample tested in 1 M NH_4_OH are about four times bigger than the ones obtained when sample was tested in 1 M KOH.

**Figure 5:**
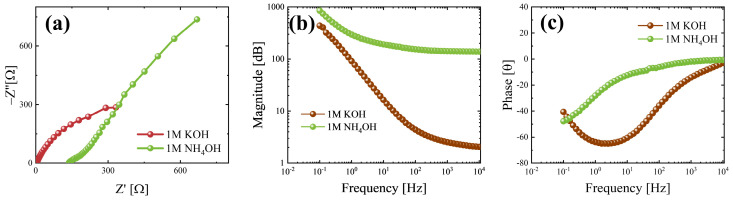
From EIS measurements, there was the possibility to evaluate (**a**) Nyquist, (**b**) magnitude, and (**c**) phase plots. The data refer to MoO_3_ nanobelts in 1M KOH (brown curve) and 1M NH_4_OH (green curve).

To deeply analyze the differences between the electrochemical behaviour in the two electrolytes, EIS measurements were performed at 0 V vs. open circuit to study the electrochemical interactions at the electrode–electrolyte interface. Nyquist plots obtained in 1 M KOH and 1 M NH_4_OH are reported in Figure 5a via the brown and green curves, respectively. The series resistance is an important parameter for electrochemical measurements, as it helps to evaluate the electrochemical resistance at the electrode–electrolyte interface, which can affect the measurements [60]. It is possible to extract the series resistance from the Nyquist plot by taking the real part of the impedance when the imaginary one approaches zero; hence, it is in the high-frequency domain. The extracted series resistances are equal to 2.5 and 140 Ω in 1 M KOH and 1 M NH_4_OH, respectively. In Figure 5b, the magnitude plot obtained in 1 M KOH and 1 M NH_4_OH is reported, as shown by the brown and green curves, respectively. In both the environments at low frequencies, high impedance values can be observed, but the green curve decreases slowly to higher frequencies, reaching a plateau close to 100 Ω after 10^0^ Hz; this behaviour suggests a pseudocapacitive behaviour [61]. On the contrary, the brown curve is characterized by a strong decrease in impedance, which can be linked to a capacitor-dominant mechanism; in this case, the plateau is for frequencies higher than 10^3^ Hz and for impedance value close to 1 Ω [61]. Moreover, the stabilization of the green curve after 10^0^ Hz indicates a resistive-dominant behaviour, while the brown one reaches stability at frequencies higher than 10^3^ Hz; hence, there is a capacitive-dominant behaviour for frequencies lower than 10^3^ Hz [62]. The sample tested in 1 M KOH has a minimum in the phase plot (see Figure 5c) between 10^0^ and 10^1^ Hz, which means that capacitor behaviour occurs at these frequencies [62], confirming that seen in the magnitude plot. The green curve (obtained when the sample was tested in 1 M NH_4_OH) stabilizes close to the zero phase very quickly with respect to the brown one; hence, the sample in 1 M NH_4_OH starts to be resistive before that in 1 M KOH [62], suggesting a possible link between lower specific capacitance values and resistive behaviour in different hydroxide environments, especially at higher scan rates.

A fitting of the EIS data was performed using the Randle circuit (see Figure S9), finding an important difference not only between the series resistances but also between the charge-transfer resistance. Indeed, the material in 1 M KOH reports a charge-transfer resistance of about 550 Ω, suggesting that the dominant storage mechanisms are linked to the formation of an electrochemical double-layer; on the contrary, the behaviour in 1 M NH_4_OH, a ten-times-lower R_ct_ was evaluated, about 50 Ω, probably due to the favoured redox reactions in this electrolyte.

A comparison with other works reporting MoO_3_-based electrodes in alkaline electrolytes is given in Table 1. Comparable potential windows were employed, but a significant improvement in the specific capacitance was registered, and a remarkable improvement in the specific capacitance can be seen.

Analyzing the shape of the CV curves, the sample clearly has a pseudocapacitive behaviour in 1 M NH_4_OH, on the contrary, in 1 M KOH, it seems to have a blended supercapacitor–pseudocapacitor behaviour; this difference was also highlighted by the shape of the GCD curves. Moreover, the sample shows high resistance in both the electrolytes, especially in 1 M NH_4_OH, explaining the low specific capacitance values at high scan rates and current densities, suggesting that its application in 1 M KOH is favourable due to the lower resistance behaviour.

## 4. Conclusions

In this work, recycled material was employed to synthesize nanostructures active for energy storage applications using a low-cost method, offering a potential approach to merge energy demand with waste management.

Orthorhombic molybdenumtrioxide (α-MoO_3_) nanostructures were obtained starting from Mo-based powder derived from industrial waste. The nanostructures, measuring 50–200 nm in width and 10 µm long, were labelled as nanobelts. The synthesis involved a pH-controlled hydrothermal process conducted at 180 °C for 3 h.

The electrochemical performance of the α-MoO_3_ was studied in two different hydroxide environments, 1 M KOH and 1 M NH_4_OH. The shape of the CV curves was analyzed, showing a pseudocapacitor behaviour for the material in 1 M NH_4_OH, while the α-MoO_3_ nanobelts showed a supercapacitor behaviour in 1 M KOH. The sample reached about 300 and 100 Fg^−1^ at 5 mVs^−1^ in 1 M KOH and 1 M NH_4_OH, respectively. The different behaviours of the material in the two environments were also investigated using GCD curves. The cup-like shape obtained in 1 M NH_4_OH confirms that the sample acts as a pseudocapacitor, while in 1 M KOH, it works as a supercapacitor. The specific capacitance extracted by the curves was about 50 Fg^−1^ for both electrodes, suggesting that the electrochemical performance was limited by the electrochemical resistance at the electrode–electrolyte interface.

Furthermore, EIS measurements were recorded to study the electrochemical processes, revealing high resistance values for both the environments. However, magnitude and phase plots suggested that in 1 M NH_4_OH, there is superior resistance behaviour than in 1 M KOH for the material. Thus, this work presents a sustainable approach to synthetize α-MoO_3_ nanobelts, which can act as pseudocapacitors and supercapacitors depending on the electrolyte environment.

## Figures and Tables

**Table 1:** Comparison between synthesized material and other MoO_3_-based electrodes in alkaline electrolytes.

Electrode Material	Electrolyte	Voltage Window [V]	Specific Capacitance [Fg^−1^]	Reference
α-MoO_3_ nanobelts	6 M KOH	0 to 1	136	[30]
α-MoO_3_ nanobelts	3 M KOH	0 to 0.65	206	[31]
MoO_3_-MWCNTs	1 M NaOH	−0.75 to 0.3	98	[63]
α-MoO_3_ nanobelts	1 M KOH	−0.6 to 0.1	290	This work
α-MoO_3_ nanobelts	1 M NH_4_OH	−0.6 to 0.1	100	This work

## Data Availability

The data presented in this study are available on request from the corresponding author.

## Supplementary Materials

The following supporting information can be downloaded at: https://www.mdpi.com/article/10.3390/nano15171380/s1, Figure S1: Low-magnification Mo-based recycled powder; Figure S2: Thanks to SEM images of the MoO_3_ nanobelts, the sample’s average size, length (a), and width (b) were studied; Figure S3: XRD pattern of the starting P-MOOX powder; Figure S4: Normalized transmittance of MoO_3_ NBs; Figure S5: CV from first to the thirtieth cycle of MoO_3_ nanobelt electrode in 1M KOH (a) and 1M NH_4_OH (b) at 20 mVs^−1^; (c) the specific capacitance values are reported with the increase in the cycle numbers; Figure S6: Cyclic voltammetry (CV) curves at different scan rates in 1M KOH (a) and 1M NH_4_OH (b); Figure S7: The calculations of Dunn’s model were performed in both the electrolytes in (a) 1M KOH at −0.25V vs Ag/AgCl and (b) 1M NH_4_OH at 0 V vs Ag/AgCl; Figure S8: Galvanostatic charge–discharge (GCD) measurements for 1M KOH (a) and 1M NH_4_OH (b); iR drop evaluation from different GCD curves at different current densities; (c) iR drop values extracted in 1M KOH (brown curve) and 1M NH_4_OH (green curve); Figure S9: The Randle circuit was employed to perform fitting of the EIS data; here, the fitted curves (red) are reported against the experimental (black) ones, with the fitting parameters reported in the attached table, both in (a) 1M KOH and (b) 1M NH_4_OH; Table S1: Visible peaks in XRD pattern; Table S2: Calculation of d-spacing for most important peaks in XRD pattern.

## Abbreviations

The following abbreviations are used in this manuscript:

CV	Cyclic Voltammetry
GCD	Galvanostatic Charge Discharge
EIS	Electrochemical Impedance Spectroscopy
EES	Electrochemical Energy Storage
TMOs	Transition Metal Oxides
CRMs	Critical Raw Materials
P-MOOX	Recycled Molybdenum Powder
PVDF	Polyvinylidene Difluoride
XRD	X-Ray Diffraction
SEM	Scanning Electron Microscope
ICDD	International Center Diffraction Data
