# Supplementary material for: MoO3 Nanobelts Synthesized from Recycled Industrial Powder and Applied as Electrodes for Energy Storage Applications
Source: Nanomaterials (Basel). 2025 Sep 8;15(17):1380. doi: 10.3390/nano15171380 (PMC12430176; doi:10.3390/nano15171380)
Supplement: Supplementary file 1 [file nanomaterials-15-01380-s001.zip › nanomaterials-3762791-supplementary.pdf]

MoO_3-Nanobelts synthesized from recycled industrial powder and applied as electrode for energy storage applications

A. Di Mauro[1], F. Ursino[1,2], G. Mineo[1], A. Terrasi[1,2] and S. Mirabella[1,2]*

1 Dipartimento di Fisica e Astronomia “Ettore Majorana”, Università degli Studi di Catania, via S. Sofia 64, 95123 Catania, Italy.

2 CNR-IMM, Università di Catania, via S. Sofia 64, 95123 Catania, Italy.

*E-mail corresponding: salvo.mirabella@dfa.unict.it

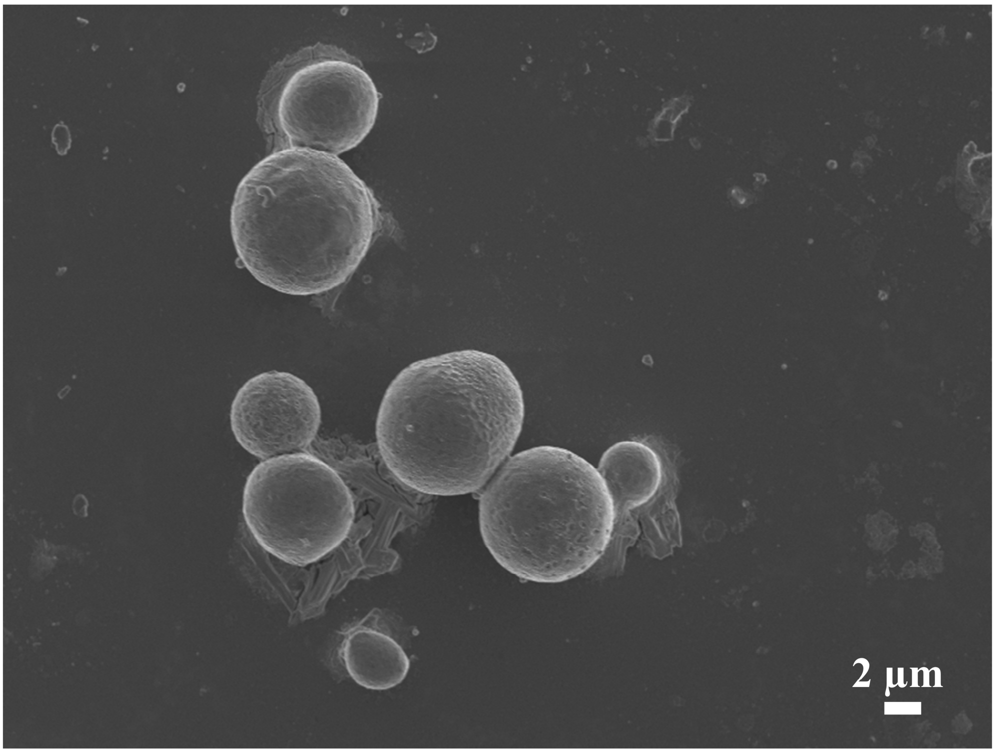

Figure S1. Low magnification Mo-based recycled powder.

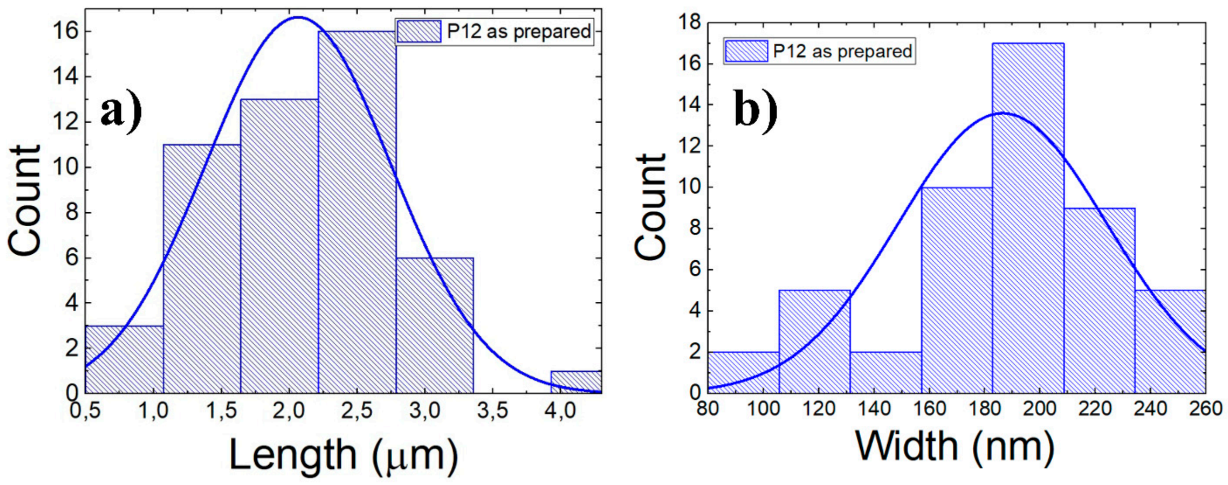

Figure S2. Thanks to SEM images on MoO_3 nanobelts sample was done an average of its size, lengths (a) and width (b) were studied.

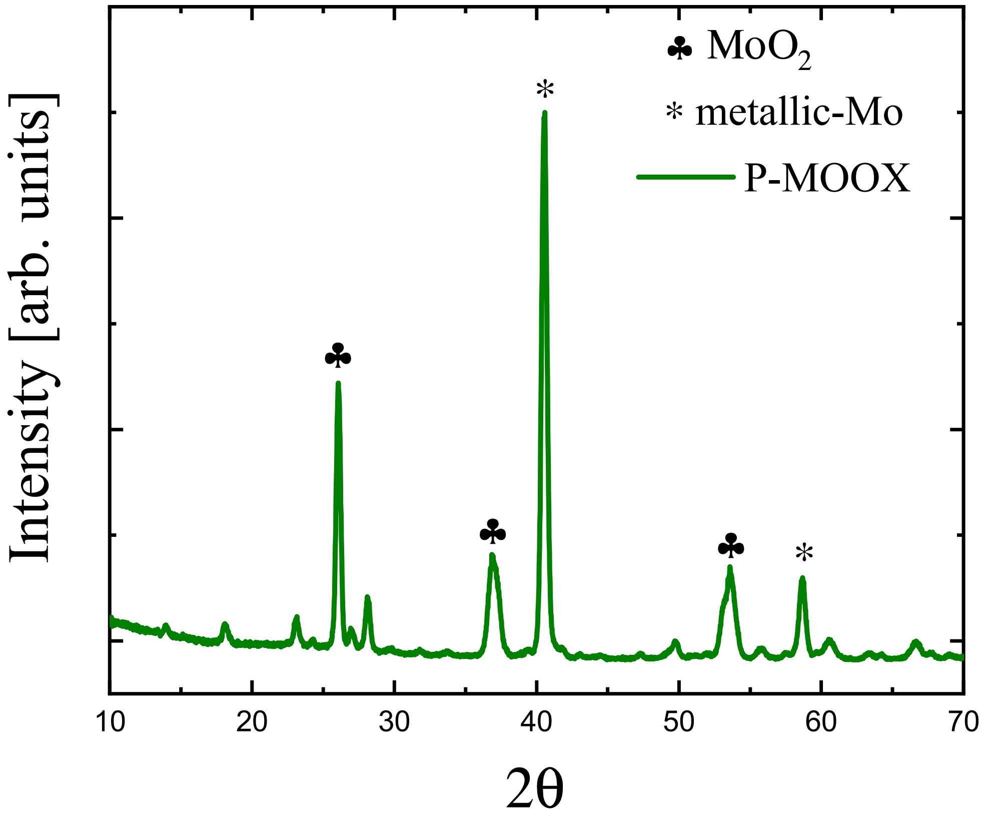

Figure S3. XRD pattern of the starting P-MOOX powder

ICDD Data 2θ (deg)	Present work 2θ (deg)	[h,k,l]	Structure	I/I_{max} ICDD	I/I_{max}
**12.76**	12.76	[0,2,0]	orthorhombic	0.34	0.27
**23.33**	23.34	[1,1,0]	orthorhombic	0.82	0.15
**25.70**	25.70	[0,4,0]	orthorhombic	0.61	1
**27.34**	27.30	[0,2,1]	orthorhombic	1	0.11
**29.70**	29.70	[1,3,0]	orthorhombic	0.13	0.09
**38.98**	39.00	[0,6,0]	orthorhombic	0.31	0.6
**45.74**	45.86	[2,0,0]	orthorhombic	0.13	0.09
**45.90**	45.86	[2,0,-1]	monoclinic	0.36	0.09
**52.04**	52.00	[1,6,1]	orthorhombic	0.05	0.07
**52.37**	52.00	[2,0,1]	monoclinic	0.13	0.07
**58.81**	58.81	[0,8,1]	orthorhombic	0.16	0.09
**61.62**	61.64	[2,6,0]	orthorhombic	0.05	0.05
**64.93**	64.84	[1,9,0]	orthorhombic	0.12	0.05
**69.47**	69.50	[2,0,2]	orthorhombic	0.06	0.08

Table S1. Visible peaks in XRD pattern.

Table S2. Calculation of d-spacing for most important peaks in XRD pattern.

2 Theta	Theta	d spacing (A)	Plane
23.34	11.67	3.81	(1,1,0)
12.76	6.38	6.93	(0,2,0)
25.70	12.85	3.46	(0,4,0)
27.30	13.65	3.26	(0,2,1)
29.70	14.85	3.01	(1,3,0)
45.86	22.93	1.98	(2,0,0)
61.64	30.82	1.50	(2,6,0)
67.52	33.76	1.39	(0,10,0)
58.81	29.41	1.57	(0,8,1)
69.50	34.75	1.35	(2,0,2)
64.84	32.42	1.44	(1,9,0)

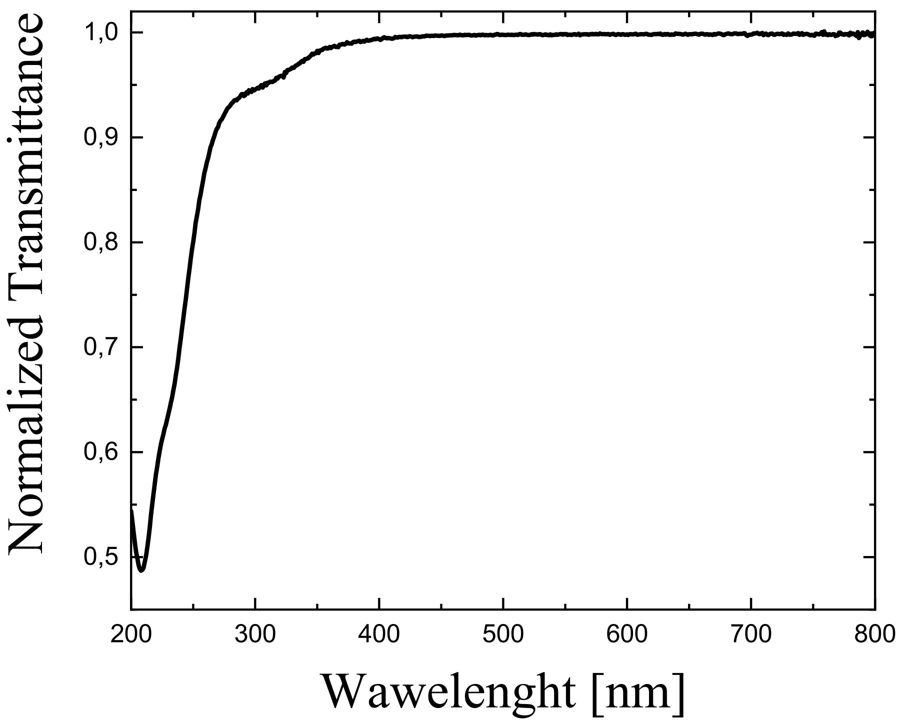

Figure S4. Normalized transmittance of MoO_3 NBs.

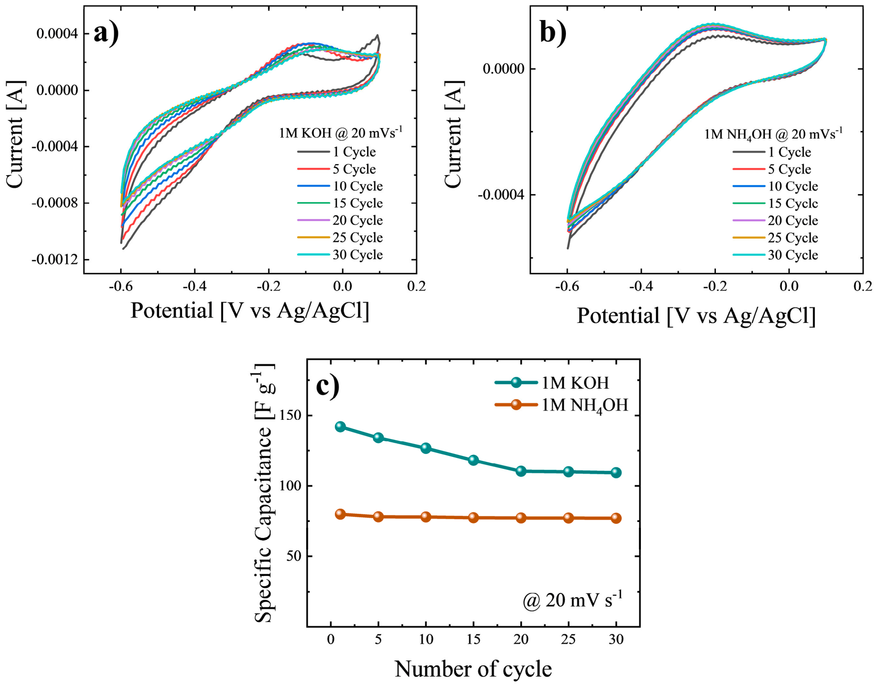

Figure S5. CV from first to the thirtieth cycle of MoO_3 nanobelts electrode in 1M KOH a) and 1M NH_4OH b) at 20 mVs^{-1}; c) the specific capacitance values are reported increasing the cycle numbers.

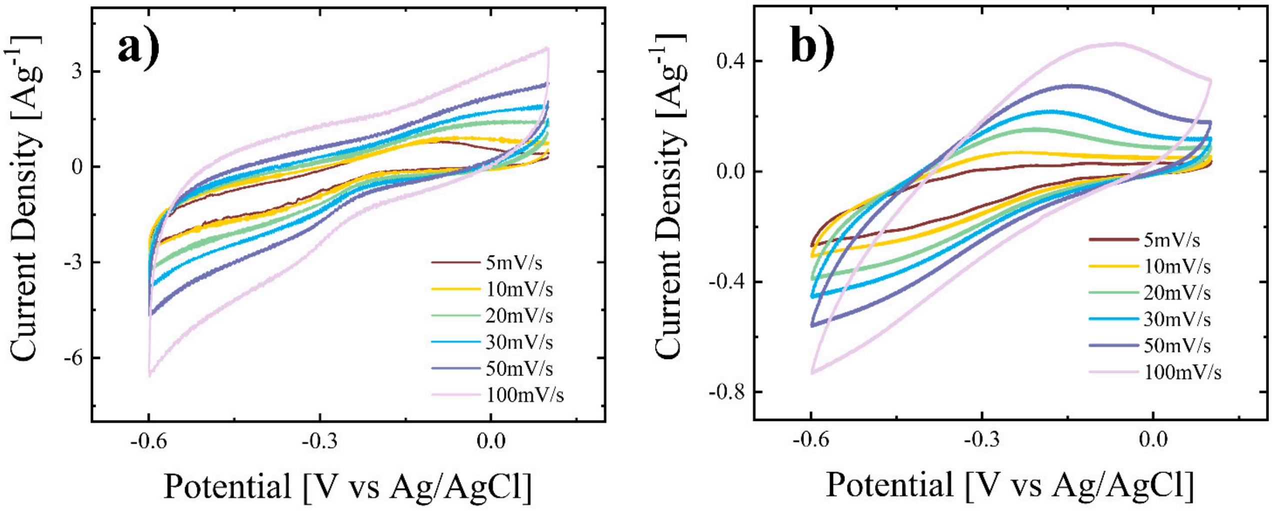

Figure S6. Cyclic Voltammetry (CV) curves at different scan rates in 1M KOH (a) and 1M NH_4OH (b).

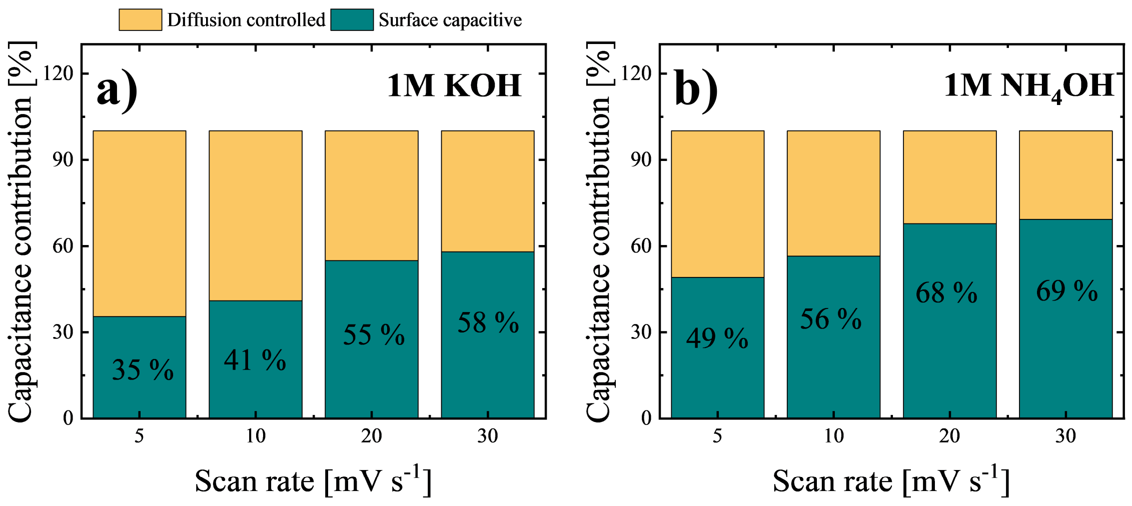

Figure S7. The calculations of the Dunn's model were performed in both the electrolytes in a) 1M KOH at -0.25V vs Ag/AgCl and b) 1M NH_4OH at 0 V vs Ag/AgCl.

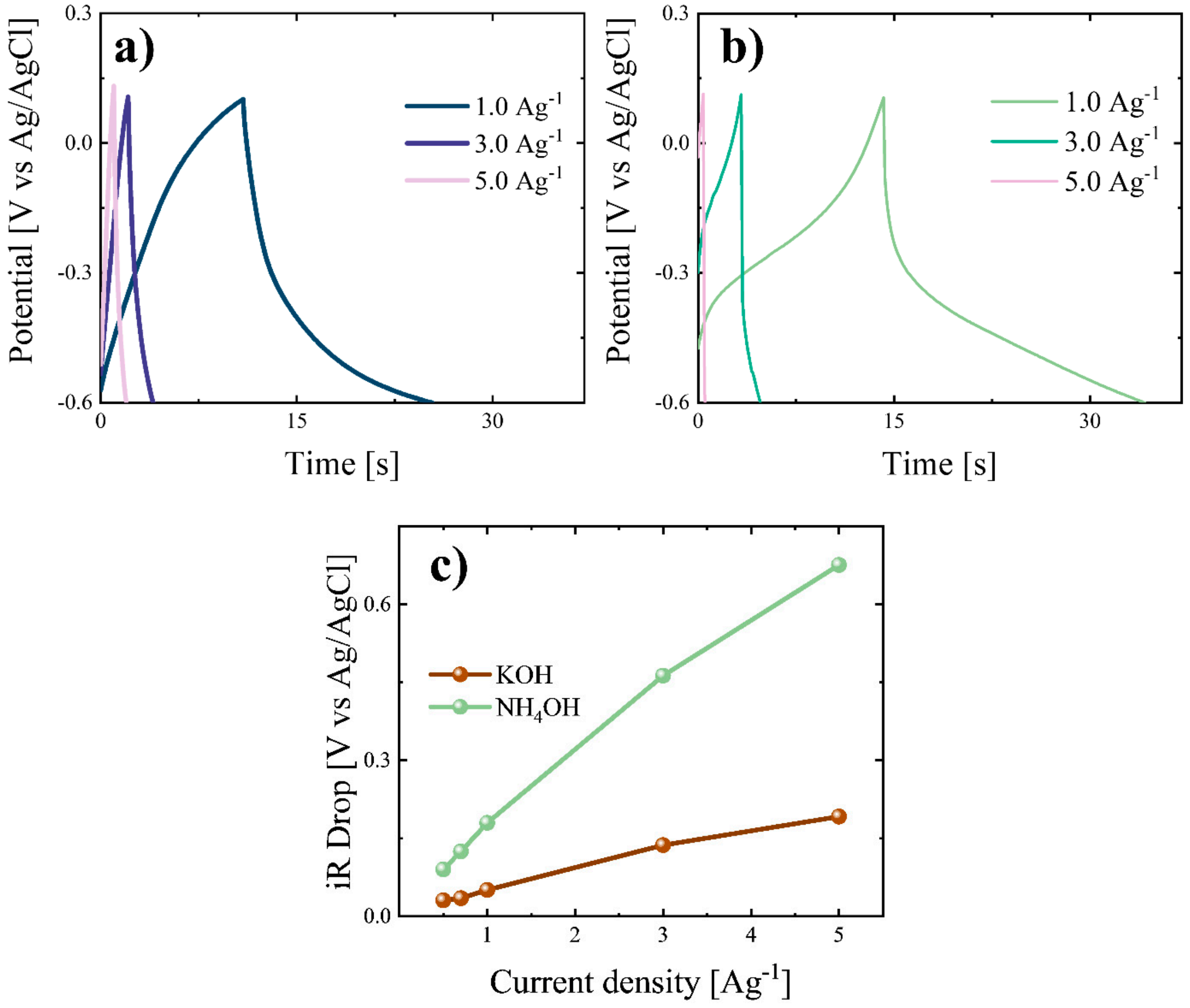

Figure S8. Galvanostatic Charge-Discharge (GCD) measurements for 1M KOH (a) and 1M NH_4OH (b); iR Drop evaluation from different GCD curves at different current density; c) iR Drop values exrttacted in 1M KOH (brown curve) and 1M NH_4OH (green curve).

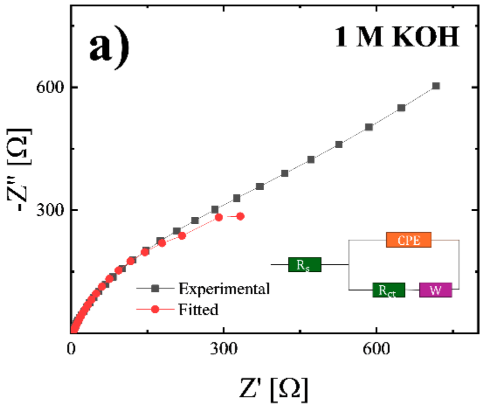

1M KOH

Circuit element	Value	Error %
**Rs**	1.96	79.13
**Rct**	550	28.52
**W**	450	100.3
**CPE**	5e-4	4.2
**n**	0.8	1.4

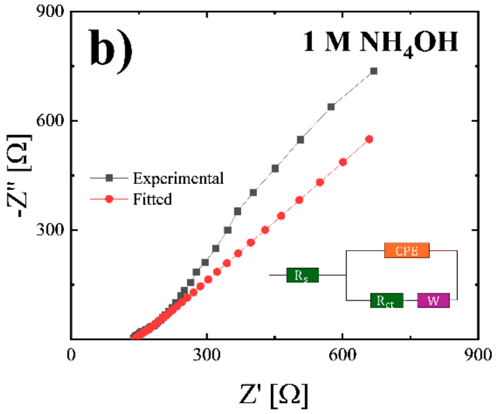

1M NH_4OH

Circuit element	Value	Error %
**Rs**	140	1
**Rct**	50	12.1
**W**	600	2.4
**CPE**	6e-4	2.8
**n**	0.6	1.7

Figure S9. The Randle circuit was employed to perform a fitting of the EIS data, here are reported the fitted curves (red) against the experimental (black) ones with the fitting parameters reported in the attached table, both in a) 1M KOH and b) 1M NH_4OH.
